# 

**Published:** 1878-07

**Authors:** 


### Contents of July Number.

### ORIGINAL.

ART. I.—Urea, and the methods employed for its Quantitative Determination. By Willis G. Tucker, M. D. ...... ............ ....... 441

ART. II.—A Case of Epiphysial Fracture of Humerus, with Dislocation. By F. Peterson. Removal of head by Dr. J. Cronyn,.... ............ 449

American Medical Association,.......... .............................. 451
Association of American Medical Colleges,..................... .. ..... 467
Convention of American Medical Editors,.............................. 470

### MISCELLANEOUS.

Catgut Sutures in Surgical Operations,................................ 472
Hippocrates Redivivus.................................................. 473
The Coming Duties of the Accoucher,............. .................. 473

### EDITORIAL.

American Medical Association,........................................ ... 476

BOOKS REVIEWED:

Ziemssen's Cyclopœdia, Vol. XIV. Diseases of the Nervous System, ............... ................................ ...... 478
Studies in Pathological Anatomy. F. Delafield,................ 478
Injuries to the Eye, and their Medico-Legal Aspect,............ 479

Notes,.... ............ .. ............................................ 479

Books and Pamphlets Received.......................................... 480

